# The Contribution of Genetic Factors to Cognitive Impairment and Dementia: Apolipoprotein E Gene, Gene Interactions, and Polygenic Risk

**DOI:** 10.3390/ijms20051177

**Published:** 2019-03-07

**Authors:** Jialing Fan, Wuhai Tao, Xin Li, He Li, Junying Zhang, Dongfeng Wei, Yaojing Chen, Zhanjun Zhang

**Affiliations:** 1 State Key Laboratory of Cognitive Neuroscience and Learning, Beijing Normal University, Beijing 100875, China; jialingfan@foxmail.com (J.F.); twh621@foxmail.com (W.T.); emmalee4539@163.com (X.L.); 2 BABRI Centre, Beijing Normal University, Beijing 100875, China; her2003@163.com (H.L.); zhangjuny1985@163.com (J.Z.); weidongfeng@aliyun.com (D.W.); 3 Institute of Basic Research in Clinical Medicine, China Academy of Chinese Medical Sciences, Beijing 100700, China

**Keywords:** Alzheimer’s disease, *APOE*, cognition, brain structure, brain function, polygenic risk score

## Abstract

Alzheimer’s disease (AD) is a progressive neurodegenerative disease. Although it has been studied for years, the pathogenesis of AD is still controversial. Genetic factors may play an important role in pathogenesis, with the apolipoprotein E (*APOE*) gene among the greatest risk factors for AD. In this review, we focus on the influence of genetic factors, including the *APOE* gene, the interaction between *APOE* and other genes, and the polygenic risk factors for cognitive function and dementia. The presence of the *APOE* ε4 allele is associated with increased AD risk and reduced age of AD onset. Accelerated cognitive decline and abnormal internal environment, structure, and function of the brain were also found in ε4 carriers. The effect of the *APOE* promoter on cognition and the brain was confirmed by some studies, but further investigation is still needed. We also describe the effects of the associations between *APOE* and other genetic risk factors on cognition and the brain that exhibit a complex gene–gene interaction, and we consider the importance of using a polygenic risk score to investigate the association between genetic variance and phenotype.

## 1. Introduction

### 1.1. Alzheimer’s Disease and Genetics

Alzheimer’s disease (AD) is a chronic progressive neurodegenerative disease characterized by memory loss and deficits in other cognitive abilities and is the most common form of dementia. With the increase in life expectancy, the prevalence of dementia rose gradually in recent years. The World Alzheimer Report 2018 estimated that, around the world, there will be one new case of dementia every three seconds; 50 million people worldwide are living with dementia in 2018, and that number will be more than 152 million by 2050. The total estimated worldwide cost of dementia in 2018 was one trillion United states dollars (US$), and this figure will rise to two trillion US$ by 2030 [1]. Based on the age of onset of the disease, AD can be divided into early-onset AD (EOAD) and late-onset AD (LOAD). EOAD, also known as familial AD, is an autosomal dominant disorder with onset before the age of 65 years, and it accounts for approximately 5% of all AD cases [2,3]. Most cases of EOAD are caused by mutations in the amyloid precursor protein (*APP*), presenilin 1 (*PSEN1*), and presenilin 2 (*PSEN2*) genes [4,5,6]. LOAD, also known as sporadic AD, accounts for most AD cases. Evidence from a twin study showed that the heritability for AD was 60–80% [7]. Genome-wide association studies (GWAS) identified susceptibility loci for LOAD, including the apolipoprotein E (*APOE*) gene, as well as the clusterin (*CLU*), phosphatidylinositol-binding clathrin assembly protein (*PICALM*), complement receptor 1 (*CR1*), bridging integrator 1 (*BIN1*), sortilin-related receptor L (*SORL1*), and translocase of outer mitochondrial membrane 40 (*TOMM40*) genes [8,9,10,11]. These genes may affect the risk of LOAD through different pathways, such as cholesterol metabolism, immune system function, and endocytic processes [12]. Among them, the *APOE* gene is the strongest risk factor for LOAD.

**Figure 1:**
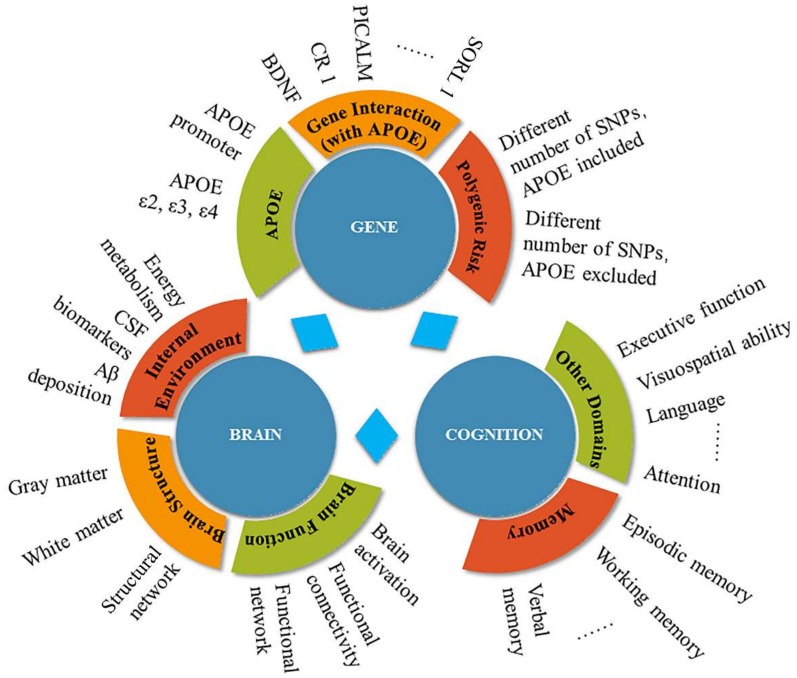
An overview of the gene, brain, and cognition facets of the current review.

Based on the *APOE* gene-related research over the last decade, we conducted a brief review focused on the influence of genetic factors, including the *APOE* gene, the interaction between the *APOE* gene and other genes, and polygenic risk factors for AD development, cognition, brain structure, and function (Figure 1).

### 1.2. APOE: Risk Factor for AD

**Figure 2:**
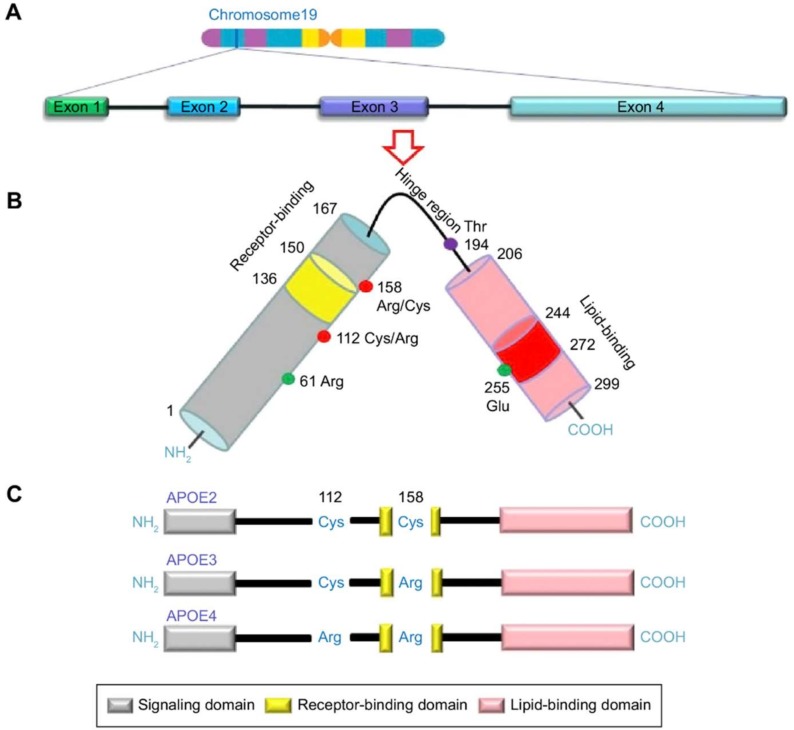
Schematic illustration of structural and functional regions of apolipoprotein E (APOE). (**A**) Location and structure of the *APOE* gene on chromosome 19. (**B**) APOE protein. (**C**) Three major APOE isoforms. (adapted from Reference [16]).

The *APOE* gene is located on chromosome 19q13.2 with a total of 3597 bases with four exons and three introns, and the gene has three major alleles (ε2, ε3, and ε4). The ε3 allele is the most frequently occurring allele, constituting 60–90% of the allelic variation, while the frequencies of ε2 and ε4 are 0–20% and 10–20%, respectively [13,14,15]. Genetic variation at the *APOE* locus induces three common isoforms: APOE2 (Cys112, Cys158), APOE3 (Cys112, Arg158), and APOE4 (Arg112, Arg158) which are coded by the ε2, ε3, and ε4 alleles, respectively (Figure 2) [14,16]. *APOE* ε4 is an acknowledged genetic risk for AD [12]. A meta-analysis demonstrated that, for *APOE* ε4 carriers, prevalence of AD was 48.7%, and homozygote prevalence was 9.6%. There are also differences across regions/ethnic groups, with the lowest ε4 carrier prevalence observed in Asia (41.9%) and southern Europe (40.9%), while northern Europe has the highest prevalence of ε4 carriers (61.3%) [17]. Each copy of the ε4 allele increases the risk of AD by approximately threefold, and two copies increase the risk of AD by 8–14-fold compared to the ε3/ε3 genotype. Conversely, the *APOE* ε2 allele has a protective effect. The risk of AD of ε2 allele carriers is only 0.6 that of the ε3/ε3 genotype [18,19,20,21]. Additionally, the age of onset of AD is also influenced by the number of *APOE* ε4 alleles and decreases by approximately 3–4 years for every ε4 allele carried [18,19]. Mild cognitive impairment (MCI) is a transitional zone between normal aging and AD, and the annual rate of progression from MCI to AD is more rapid than the progression to AD of normal subjects [22]. Meta-analysis also revealed that the *APOE* ε4 allele was associated with more than double the risk for progressing from MCI to AD across studies compared to ε4 non-carriers [23,24]. Thus, *APOE* ε4 is associated with developing AD by increasing the relative risk of AD and by lowering the age of onset of the disease. Other rare *APOE* variations in addition to the three common alleles were also reported [25,26]. For example, the ε7 allele, with two lysine residues replacing glutamic acid at positions 244 and 245 in the carboxyl terminus, is associated with hyperlipidemia and atherosclerosis [27]. Furthermore, since the ε7 mutant (like ε4) associates preferentially with very low-density lipoproteins, it is also presumed to be related to AD risk. Youn and colleagues investigated the association between *APOE* ε7 expression and cognitive impairment, with the results suggesting that ε7 could serve as a risk factor for cognitive impairment and is particularly associated with vascular disease [28]. However, related research is still limited, and further studies are needed to investigate the role of ε7 in cognitive impairment and AD.

In addition to polymorphisms within the coding region, the polymorphisms within the *APOE* promoter region are also related to AD risk [29]. These polymorphisms are proposed to modulate the transcriptional activity of the *APOE* coding region [30]. Three single-nucleotide polymorphisms (SNPs) (491A/T, rs449647; 427T/C, rs769446; 219T/G, rs405509) within this region were identified [31]. Xin and colleagues conducted a meta-analysis of 40 studies with 9662 cases and 9696 controls, and revealed that the rs449647 polymorphism and rs405509 polymorphism showed a modest but significant association with AD susceptibility, identifying rs449647 AA and rs405509 TT as risk factors. However, the association between the rs769446 polymorphism and the risk of AD is not consistent, as a significant association was not identified in this meta-analysis [32]. Nonetheless, a recent meta-analysis of 23 publications that included 5703 AD patients and 5692 controls revealed that the C allele of rs769446 was significantly associated with an increase in the risk of AD, while there was no association between the rs449647 or rs405509 polymorphisms and the risk of AD [33]. The two meta-analyses included 13 overlapping studies. The reasons for these heterogeneous conclusions may include differences among study designs, including the studied populations, sample selection (e.g., age of onset, diagnosis criteria), sample size, methods, and interactions with other risk factors, especially *APOE* ε4 status. Thus, the relationship between *APOE* promoter polymorphisms and the risk for AD may differ across studies, and represents a complex pattern with the *APOE* genotype. Future studies including large samples and different ethnicities should be conducted.

## 2. The Effects of the *APOE* Gene on Cognitive Function and Dementia

### 2.1. APOE ε4 Allele

Negative effects associated with this gene on cognition are also found in *APOE* ε4 carriers. Compared to non-carriers, *APOE* ε4 carriers exhibit worse cognitive performance and accelerated cognitive decline in healthy, MCI, and AD subjects [34,35,36].

As memory deficits are considered the major cognitive impairment in AD [37], the relationship between memory decline and the presence of the *APOE* ε4 allele in AD was examined a lot. Van der Vlies and colleagues investigated the relationship between the *APOE* ε4 allele and cognitive function in 229 AD patients, and the results indicated that memory was more impaired in ε4 carriers than in non-carriers, suggesting that the *APOE* genotype may modify the clinical phenotype of AD [38]. Similar results were also found in a study including 523 AD patients that evaluated memory performance based on standard tests and daily function, and found a strong relationship between amnestic presentation and the parameters of increased age of onset, family history, and the presence of the *APOE* ε4 allele [39]. Wolk and Dickerson investigated a broad range of cognitive functions, including verbal memory, working memory, executive function, and verbal ability, and they found that the ε4 carriers exhibited decreased verbal memory and increased medial temporal lobe (MTL) atrophy compared with the non-carriers who exhibited poor working memory, executive function, and verbal ability, as well as increased frontoparietal atrophy [40]. Similar results were also found by Kim and colleagues when examining the impact of the *APOE* ε4 allele in AD patients of different ages. AD patients under 75 years of age who did not carry the *APOE* ε4 allele or who were heterozygotic showed poor performance in language, visuospatial, and frontal functional tests, while homozygotes older than 75 exhibited worse memory. The cortical atrophy pattern was also different across genotypes and age groups [41]. A longitudinal study also indicated that the ε4 allele predicted a faster cognitive decline than other alleles, with gene dose-dependent effects observed in mild AD [42] and more evident in younger AD patients than in older AD patients [43]. Taken together, these results suggest that AD patients who are ε4 allele carriers and non-carriers may suffer from different patterns of cognitive dysfunction, in which age may play an important role.

Farlow and colleagues investigated the impact of the *APOE* genotype on MCI in 494 participants, and they found that the presence of the *APOE* ε4 allele decreased global cognition and memory performance, seemingly resembling the cognition and memory of patients in the early stages of AD [34]. Another study also found an association between the *APOE* ε4 allele and impaired memory function in both middle-aged and elderly MCI subjects [44]. Furthermore, longitudinal studies revealed that the *APOE* ε4 genotype is predictive of an increased decline in general cognition in MCI, as the ε4 carriers had a significantly more rapid decline [45] in addition to a higher conversion rate to AD, as mentioned above [23,24].

Similar to MCI or AD, the negative effects associated with this gene are also found in cognitively healthy *APOE* ε4 carriers. Although some studies indicated null negative effects [46,47], many studies indicated that, compared to non-ε4 carriers, ε4 carriers exhibit worse performance in a range of cognitive functions [36,48] at old age, especially in episodic memory [49,50,51,52], executive function [50,52,53,54], and global cognition [52,55]. In addition to cognitive status, *APOE* ε4 is also related to accelerated cognitive decline in old adults, particularly in memory [36]. Caselli and colleagues investigated the *APOE* ε4 effect on memory decline in cognitively normal subjects aged between 21 and 97 years. After approximately five years of follow-up, they found that the ε4 carriers exhibited memory decline beginning before the sixth decade of life and that memory declined more rapidly than in non-carriers [56]. Homozygous subjects had the most rapid memory decline, indicating that the *APOE* ε4 effect on cognitive decline may be dependent on gene dose [56,57]. Similar results were found in studies with different follow-up times and subjects of different ages [58,59]. Additionally, studies across ethnic groups and regions also confirmed the negative effect of the ε4 allele. Barnes and colleagues found that *APOE* ε4 is related to a more rapid rate of decline in episodic memory in blacks, similar to the effect of this allele in whites [60]. Another study also found a significant ε4 effect on the incidence of AD and on the cognitive decline in Yoruba and African Americans in a large longitudinal comparative study [61]. Lipnicki et al. conducted a longitudinal study of 14 cohorts from 12 countries to investigate the relationship between cognitive decline and the *APOE* gene, with a follow-up duration of 2–15 years. They found that ε4 allele carriers exhibited a slightly more rapid decline in cognitive function, including memory, processing speed, and language than non-carriers [62].

Although some studies did not find significant results, the detrimental effects of the *APOE* ε4 allele on cognitive function were confirmed. In addition, memory is commonly affected by the ε4 allele with gene dose-dependent effects in AD, MCI, and cognitively healthy subjects. The inconsistent null pattern in some studies may derive from methodological issues, including differences in age, cognitive measures, sample size, and other cognitive risk factors [36,48]. It is noteworthy that the differential effects of *APOE* ε4 on cognition during different life stages represents an example of antagonistic pleiotropy [63]: a reduced or null negative effect is observed in middle age [50,64]; a reversed positive effect is observed in young adults [49]; and the effect size in the elderly is also affected by age [48]. Thus, any *APOE*-related study must take into account the interaction between age and the *APOE* gene.

### 2.2. Promoter Polymorphisms of the APOE Gene

In addition to the risk for AD, polymorphisms in the *APOE* gene promoter also affect cognition in the aged. Shu and her colleagues included 837 non-dementia elderly subjects living in the community and found that participants with the rs405509 TT genotype showed worse general cognitive function, attention, and executive function than the G allele carriers, regardless of *APOE* ε4 state [65]. Similarly, Chang et al. also confirmed that the rs405509 TT genotype is significantly associated with poor general cognitive function, episodic memory, and executive function. After controlling for the *APOE* ε4 genotype, the TT genotype also had a significant age-related decline in global cognition, memory, processing speed, and executive function [66]. Poor language performance in the rs405509 TT genotype was also found in elderly subjects without dementia, and the TT genotype exhibited a more rapid rate of decline in global cognition [67]. Ma and colleagues investigated the interaction effect of the rs405509 T allele and the *APOE* ε4 allele on cognitive ability in Chinese participants, and found significant interaction effects between rs405509 and *APOE* on general cognition, memory, and attention. The double homozygous genotype (rs405509 TT/ε4ε4) exhibited a significant reduction in general cognition, memory, and attention [68]. However, in a recent study that included elderly men from Finland, researchers found that the rs405509 TT genotype was significantly associated with improved general cognition, language, arithmetic, and visual spatial ability, independent of the *APOE* major isoforms [69]. This likely indicates that the effects of rs405509 are different among different ethnicities. In addition, the researchers found that subjects with the CC genotype of rs440446 showed better visual spatial ability than subjects with the GG genotype. These studies suggested that the promoter of *APOE* can significantly affect cognitive function, but only a few studies were performed, and most of these studies focused on rs405509. Further studies including multiple promoters may increase the understanding of the effect of *APOE* on cognition.

### 2.3. Genetic Association with the APOE Gene

Although the *APOE* gene explains a part of the genetic risks associated with AD, other genes may still modify the *APOE* ε4 effect [70]. Previous *APOE*-related studies also investigated the effects of associations between *APOE* and other gene polymorphisms on cognition and dementia (Table 1).

Ward and colleagues compared cognitive function in 433 older adults to determine the association between *APOE* and brain-derived neurotrophic factor (*BDNF Val66Met*) polymorphisms. They found a significant *APOE* × *BDNF* interaction in episodic memory, with *APOE* ε2 carriers displaying episodic memory superior only in *BDNF* Met carriers [71]. Episodic memory was also found to be impaired in a sample of *APOE* ε4-carrying MCI/AD subjects who were also carriers of the Met mutation of *BDNF* compared to those who Val/Val homozygotes for *BDNF* [72]. When exploring the synergistic effects of *BDNF*, *APOE*, and HbA1c (glycated hemoglobin) on cognitive decline over 10 years in adults without dementia, Persson et al. found joint effects on memory decline in *BDNF* × *APOE* × age, with the subjects carrying the Met allele, as well as at least one copy of the *APOE* ε4 allele, showing magnified effect sizes with increasing age on memory decline, while the homozygote Val subjects carrying the ε4 allele showed a decreased slope [73]. *APOE* and *BDNF* may impact cognition together through their interactional effect, but this effect is impacted by education because neither *APOE* nor *BDNF* modify the beneficial effects of a university-based educational intervention on cognitive function [74].

Martinez and colleagues examined the synergistic effects of the catechol-*O*-methyltransferase (*COMT* rs4680) gene and *APOE* in AD and MCI subjects. Although neither *COMT* alleles nor genotypes were independent risk factors for AD or MCI, the high-activity genotypes increased the risk of AD in ε4 carriers and exhibited a synergistic interaction with the *APOE* allele. *COMT* heterozygotes who carry the ε4 allele exhibited a higher risk for MCI [70]. This study is in accordance with findings by Wang et al., in which the subjects with the *COMT* high-activity genotypes had an increased risk of developing AD with the presence of at least one ε4 allele [75]. Additionally, the combined *COMT* and *BDNF* risk is also associated with poor executive function in ε4 carriers without dementia [76].

The *TOMM40* gene shows a linkage disequilibrium pattern with *APOE* and is associated with the development of AD [77]. Longer poly-T tracts at *TOMM40* rs10524523 (‘523) are significantly correlated with earlier age of onset of LOAD in *APOE* ε3 carriers [78,79]. Johnson and colleagues investigated the relationship between *TOMM40*’523 and cognitive function in 117 *APOE* ε3 homozygous adults, and found that those who were homozygous for very long (VL) poly-T lengths had poorer memory than those who were homozygous for short (S) poly-T length [80]. However, another study also found that the S/S poly-T genotype with *APOE* e3/3 homozygosity was related to more rapid declines in global cognition and memory in community-based older persons [81]. Thus, the effect of *TOMM40* on cognition and whether there are additive effects between *APOE* and *TOMM40* require further investigation.

The effect of the *APOE* gene on cognition and dementia is also modified by other genetic factors, including the *SORL1* [82], *PICALM* [83,84], *CR1* [83,85], *ABCA7* [86], *TREM2* [87,88], and *BIN1* [83,84], showing complex gene-gene effects. Thus, combining multiple genes and investigating their joint effects is a logical experimental design and may help clarify these complex genetic processes.

### 2.4. Polygenic Risk Factors for Cognitive Decline

As cognition and AD are highly heritable polygenic traits in humans, investigating polygenic effects on phenotypes is also very important. Therefore, researchers tend to integrate different genetic loci to construct polygenic risk scores (PGS). Studies confirmed that polygenic genetic risk increases the incidence of AD [89], increases the conversion rate of MCI to AD [90], and increases the risk of cognitive decline [91,92,93].

A study of older adults in Belgium showed that weighted PGSs consisting of 22 SNPs (including *APOE* ε4) increased the incidence of AD 2.32-fold, and the age of onset of AD decreased by 2.39 years per unit increase in risk score [89]. Another study in the Han Chinese population also found PGSs based on three SNPs were associated with AD risk independent of *APOE* genotype, and PGSs based on AD risk-associated SNPs may supplement APOE for better assessing individual risk for AD [94]. Marden and colleagues also found that the PGSs based on ten polymorphisms confirmed to predict AD can predict dementia risk among both non-Hispanic whites and blacks [92]. Lee and colleagues generated AD risk prediction models using a combination of the top-ranked SNPs associated with AD in a Korean elderly sample; when considering age effect, their models were able to predict the onset of AD in an independent Japanese AD sample, suggesting the potential practical clinical use of combining age and polygenic risk score in predicting AD [95]. Therefore, the association of polygenic risk score and the incidence of AD was confirmed in different ethnic groups. In addition, increased polygenic risk score can also increase the conversion rate of MCI to AD. A study that included eight risk SNPs (*APOE* excluded) found that, when an individual carried six or more risk alleles, the conversion rate of MCI to AD increased twofold [90]. Another European multicenter study found that weighted PGSs constructed with nine AD risk SNPs were significantly associated with the conversion of MCI to AD in *APOE* ε4 allele carriers [96]. A study of MCI patients also found that weighted PGSs calculated with 18 non-*APOE* AD risk variants were significantly associated with a decline in general cognition [97].

Verhaaren and colleagues included 5171 middle-aged and older people without dementia to investigate the association of weighted PGSs constructed with 12 AD risk SNPs (including *APOE* ε4) with general cognition, memory, and possessing speed. A significant correlation between PGS and memory was found, and this association was attenuated when the *APOE* ε4 allele was excluded [91]. Similar results were also found in an Australian study on non-demented elderly, and the study found that the weighted PGSs using 12 risk SNPs (including *APOE*) were related to episodic memory [98]. Marden and colleagues included non-Hispanic white and black participants and examined the efficacy of weighted PGSs constructed by 10 AD risk SNPs (including *APOE*) in predicting AD and memory performance. The study found that PGSs were associated with a risk of dementia among whites and blacks, whereas the association between PGS and poor memory performance was only found in whites [92]. Similar results were also found in a longitudinal study. In 2016, researchers included a total of 8253 non-Hispanic white and black participants and examined the interaction of weighted PGSs constructed by 22 AD risk SNPs (including *APOE*) and age to predict memory decline. The study found that PGSs with the *APOE* ε4 allele and PGSs without the *APOE* ε4 allele in whites both can predict a more rapid decline in memory, whereas only PGSs with the *APOE* ε4 allele in blacks can predict a faster decline in memory, indicating the genetic efficacy of *APOE* in different ethnic groups [93]. Carrasquillo et al. also found that PGSs including the *APOE* ε4 allele were associated with memory decline and the progression to MCI/AD in a Caucasian cohort [99]. PGSs are an appropriate method to integrate multiple genetic risk factors. As PGS is an emerging method, the number of studies is also limited. Future related studies may help deepen our understanding of genetic effects on cognitive function and dementia.

## 3. The Effects of the *APOE* Gene on Brain Function

### 3.1. APOE ε4 Allele

The β-amyloid (Aβ) hypothesis is the most accepted pathological theory of AD, and it is believed that Aβ deposition causes a series of subsequent pathological changes, including the emergence of p-tau and the increase in neurofibrillary tangles in the brain. A number of previous reviews summarized a significant correlation between *APOE* **ε**4 and amyloid load in the brain [2,100,101]. We overviewed the articles investigating the influence of *APOE* **ε**4 on brain amyloid deposition published in the last decade, and this conclusion is well proven. Pittsburgh Compound-B (PiB) uptake in the temporoparietal and frontal cortices of patients with AD is positively correlated with **ε**4, and both the distribution and the annual increase in Aβ deposition exhibited a gene dose-dependent effect [102,103]. The same relationship is observed in patients with MCI and in cognitively healthy elderly subjects; those who carry **ε**4 have more deposition than non-carriers [104,105]. In fact, a meta-analysis summarized by Jansen and colleagues revealed Aβ deposition onset early in the fourth decade of life of **ε**4 carriers, and they had Aβ deposition levels that were two to three times higher than that of non-carriers [101]. *APOE* **ε**4 also reduced the age that Aβ was detected; Aβ positivity occurs in normal subjects at approximately 56 years of age and in non-carriers at approximately 76 years of age [106]. A significant *APOE* × age interaction was also observed by Gonneaud and colleagues. Aβ deposition increased nonlinearly with age in *APOE* **ε**4 carriers but not in non-carriers in their study [107]. Moreover, it seems that the association between Aβ deposition and cognitive performance is modified by *APOE* status. One study reported that higher amyloid content was predictive of a longitudinal decline in executive function and memory tests in **ε**4 non-carriers [108]; however, another study reported severe cognitive impairment in addition to higher Aβ deposition in **ε**4 carriers [109]. This discrepancy may result from the difference of age in the two groups (62 years old in the first study and 80 years old in the latter one). Furthermore, there was also a report that increased cognitive activity over a lifetime can diminish cortical PiB retention in **ε**4 carriers [110], which makes the relationship among *APOE*, cognition, and Aβ deposition quite interesting and worthy of further study.

Corresponding to the influence of **ε**4 on Aβ deposition, a study also showed that the decrease in Aβ_42_ and the increase in tau/p-tau in the cerebrospinal fluid (CSF) were related to **ε**4. Liu and colleagues observed 1718 participants from Alzheimer’s Disease Neuroimaging Initiative (ADNI) and found significantly decreased Aβ_42_ and increased tau/p-tau levels in the CSF in the **ε**4 carriers, which appeared earlier than other biomarkers of AD [111]. This effect is gene dose-dependent; homozygosity for the *APOE* **ε**4 allele results in a worse phenotype than both heterozygosity and lack of the *APOE* **ε**4 allele. The influence of **ε**4 on CSF biomarkers was even stronger than the clinical status [112,113]. However, the association between tau and the *APOE* genotype is not observed in people without Aβ [114,115]. Considering the negative correlation between Aβ deposition in the brain and Aβ_42_ in the CSF, as well as the influence of Aβ deposition on the change in tau/p-tau, this relationship between **ε**4 and the CSF biomarkers is understandable. However, there are negative results that should arouse our attention at the same time. For example, a study including samples from Sweden, Finland, and Germany found that the change in CSF Aβ_42_ was independent of the *APOE* genotype in some of their sub-cohort. They also found that **ε**4 may not have a direct effect on CSF levels of Aβ_42_, but did demonstrate *APOE* **ε**4-associated preclinical pathology in the elderly [116]. For this reason, the effect of **ε**4 on CSF Aβ_42_ may not exist in some younger sample populations because they do not have amyloid.

As one of the earliest biomarkers of AD, glucose hypometabolism is also affected by the *APOE* genotype. Dozens of studies observed that preclinical **ε**4 carriers have glucose hypometabolism in regions similar to the change in glucose patterns in AD [117], including regions in the parietal, temporal, and prefrontal areas, with the posterior cingulate cortex (PCC) representing the most significant region [118]. This association could be observed in cognitively normal individuals as early as 30 years of age [119]. As a risk factor, an increase in the number of **ε**4 alleles will result in worse glucose metabolism with increasing age, and this gene dose effect may be more severe in women [120]. A few researchers reviewed articles on *APOE* **ε**4 and metabolism, suggesting that the dysfunction of many brain parameters related to metabolism may reflect an inherent dysregulation of glucose metabolism in **ε**4 carriers [121]. For instance, Zhao et al. reported that **ε**4 may impair cerebral insulin signaling, which could partly explain the way **ε**4 affects glucose metabolism [122]. Moreover, the association between **ε**4 and the reduced mitochondrial cytochrome oxidase activity observed in the PCC may also contribute to this abnormal energy metabolism in the brain [123].

*APOE* can also influence brain structure to a certain extent, including the atrophy of the hippocampus, amygdala, entorhinal cortex, and total brain volume. Additionally, increases in white-matter hyperintensity volumes are often reported in many studies [124]. Among the gray-matter atrophy associated with **ε**4, the hippocampal region was the most frequently mentioned. A meta-analysis conducted by Liu and colleagues found a statistically significant association between the **ε**4 allele and hippocampal atrophy in six cross-sectional studies that included both healthy elderly subjects and MCI/AD subjects [100]. They further confirmed this association between **ε**4 and hippocampal atrophy in late MCI and AD patients, with a gene dose effect even shown in the late MCI patients [111]. Other studies also reported that increasing **ε**4 gene dose caused hippocampal atrophy [125,126]. Atrophy is not restricted to the hippocampus. A previous study also found an association of the **ε**4 allele with increased susceptibility of the temporal cortex and decreased vulnerability in the frontoparietal neocortical regions in AD patients [127,128,129]. However, at the same time, many studies showed that **ε**4 and gray-matter atrophy were not always closely related. Several studies compared the gray-matter volume of **ε**4 carriers and non-carriers and found no difference [130,131], especially in young children and elderly with advanced age [132,133]. This phenomenon makes researchers suggest that the effect of **ε**4 may not be detectable when the subjects are young, and, in old age, the shrinkage caused by age may mask the effect of **ε**4 [124]. The effect of **ε**4 on brain structure may also be an example of antagonistic pleiotropy. However, considering that reports also showed negative results in middle-aged adults [134], another suggestion is that the atrophy influenced by **ε**4 may only occur more proximal to the onset of clinical symptoms of dementia [133], which is in agreement with the close relationship between **ε**4 and AD. The study of white matter is very similar to that of gray matter, and both positive and negative results were reported. The area impaired by **ε**4 was mainly centered in regions around the medial temporal lobe, with decreased white-matter fractional anisotropy (FA) in the left parahippocampal gyrus [135], limbic, and medial temporal regions [136], decreased mean diffusivity (MD) in the corona radiate and corpus callosum, and decreased axial diffusivity (AD) in the genu of corpus callosum [137]. Since *APOE* has effects on the brain vascular system [121], it may be one of the **ε**4-mediated pathways that impairs white matter, and some cerebrovascular diseases could accompany these effects [138].

Functional MRI (fMRI) can reflect brain activity both in a resting state and while performing a task. The first fMRI study that investigated gene effects on brain function was conducted by Smith and colleagues in 1999, who reported decreased brain activation during a visual task in **ε**4 carriers [139]. Trachtenberg and colleagues reviewed 27 fMRI studies before 2012 and concluded that **ε**4 carriers can present both decreased and increased brain activation compared to non-carriers, and the location of change was inconsistent as well. They thought the reasons for these inconsistent findings regarding the direction and location of activation among studies included the different tasks used in the studies, the different family history of AD of the participants, and the age difference [140]. The **ε**4 carriers exhibited damaged connectivity between the precuneus and several regions during a memory encoding task [141], but recruited more regions in low load and displayed fewer increases in activation in high load in an n-back working task [142]. Old age was associated with increased activity in **ε**4 carriers in a face–name task [143]. Brain activity is susceptible to many factors during task fMRI; it is quite difficult to identify a consistent pattern; however, with an increasing number of resting state studies in recent years, the functional connectivity (FC) related to certain areas is more consistently affected. The default mode network (DMN) was the most affected network; both decreased FC inside the DMN [144] and decreased FC between the DMN and other networks [145] were found in different studies. FC alterations between the hippocampus or parahippocampal region and other brain regions were also frequently reported [146,147,148,149]. At the same time, there are still other areas with both increased and decreased FC characteristics reported in the literature [150,151,152]. Although we excluded the effect of task paradigm in resting fMRI, the differences in age, disease stage, and sex were still easily unified in different research samples.

### 3.2. Promoter Polymorphisms of the APOE Gene

In addition to the impacts on cognitive function and risk for AD mentioned above, polymorphisms in the promoter region of the *APOE* gene influence brain structure and function. Lambert and colleagues measured the Aβ load in Brodman areas 8 and 9 in 74 AD patients with different promoter polymorphisms. Both rs449647 AA carriers and rs405509 TT carriers showed a significant increase in the Aβ load of *APOE* **ε**4 non-carriers compared to that of carriers [153]. Another study in elderly people without dementia also found that the rs449647 AA carriers had significantly increased Aβ deposition [154]. However, at the same time, it is noteworthy that there is study a reporting conflicting results, which showed that the severity of cerebral amyloid angiopathy was not affected by rs449647 and rs405509 genotype [155]. Our recent work systematically studied the impact of rs405509 on brain structure and function. We observed that there was a significant interaction between rs405509 and *APOE* on general mental status in a sample of 836 community-based elderly people, and a significant interaction between rs405509 and *APOE* on the right inferior temporal gyrus and right fusiform gyrus in 102 people who had an MRI scan. The carriers of both **ε**4 and rs405509 T had the smallest gray-matter volume [68]. The interaction between rs405509 and age was also demonstrated in 120 people without dementia. The carriers of the rs405509 TT genotype showed a steeper decline with aging than the G carriers, and the cortical thickness covariance between several brain regions was also modulated by the interaction of the rs405509 genotype and age [67]. The same interaction effect was observed in the white-matter network; the rs405509 TT carriers had reduced global and local efficiency, mainly in the left anterior and posterior cingulate cortices [65], and decreased network betweenness centrality in the left inferior frontal gyrus pars opercularis, the left posterior cingulate cortex, the right inferior occipital gyrus, and the left angular gyrus [66]. Additionally, resting-state fMRI revealed that rs405509 also significantly interacted with *APOE* in the anterior cingulate gyrus, medial frontal region, and precuneus in the anterior and posterior DMN, with both TT and **ε**4 carriers mostly impairing the DMN [156]. The results listed above suggest that the promoter of *APOE* can significantly affect the structure and function of the brain. However, only a few studies related to APOE were performed, which limits our understanding of its importance.

### 3.3. Genetic Association with the APOE Gene

Similar to the cognitive function we mentioned above, the interaction between other genes and *APOE* can also be demonstrated with neuroimaging (Table 1). Elderly people who carry both *CHRNA4* (a nicotinic receptor subunit gene) TT and *APOE* **ε**4 showed slower reaction time and lower white-matter volume in a visuospatial attention task [157]. The risk allele of the *PICALM* gene in rs3851179 may also interact with *APOE* ε4 to cause gray-matter volume impairment of the prefrontal cortex [158]. Carrying a risk allele of the *CR1* rs3818361 results in a reduced brain amyloid burden compared to non-carriers, but only in **ε**4 non-carriers; *APOE* **ε**4 individuals show a significantly increased brain amyloid burden [159]. Liu and colleagues found that the effects of *APOE* **ε**4 on the activation of the triangular part of the right inferior frontal gyrus were modulated by rs2618516 in the spondin 1 (*SPON1*) gene in a working memory task [160]. Another study reported alterations of FC between the hippocampus and inferior frontal gyrus in a resting-state network in people who carry both the G allele of the *SORL1* gene and the **ε**4 allele of *APOE* [161]. In addition, significant *APOE* risk-allele-dependent reduction in the brain FC density in the dorsolateral prefrontal cortex can be observed in carriers of the TT allele of the kidney and brain expressed protein (*KIBRA*) gene, but a significant *APOE* risk-allele-dependent increase in the brain FC density was observed in carriers of the C allele of *KIBRA* [162]. At the same time, people with both *APOE* ε4 and *KIBRA* C were observed to have significant hippocampal atrophy [163]. In addition to the genes we described above, there are still many other genes that have significant interactions with *APOE*, and their effects on neuroimaging are also worth further exploring. All of these results suggest that different AD risk genes may interact with each other, and this interaction can cause many observable effects on neuroimaging. It is important to consider the interaction between genes when we discuss the influence of innate factors on AD pathology, and the underlying mechanism still needs to be further studied.

### 3.4. Polygenic Risk for the Brain

In recent years, polygenic risk was frequently applied to AD studies because it maximizes the impact of all genetic factors. An increasing number of studies proved that polygenic risk can cause significant changes in brain structure and function.

Sleegers and colleagues found that PGS consisting of 22 SNPs was negatively correlated with the density of Aβ_42_ in the CSF in a sample of elderly people in Belgium [89]. The same negative correlation was also found in a Dutch study with 22 SNPs, coupled with a significant correlation between PGS and the density of tau in the CSF [164]. Louwersheimer and colleagues reported that the density of tau and p-tau in CSF was significantly correlated with PGS consisting of 18 SNPs, not including *APOE*, in a group of MCI patients [97]. In another study, Voyle and colleagues used case control PGS (*APOE* excluded) to predict CSF tau and Aβ, and they found it to be more predictive of Aβ and tau pathologies than the predictors that consisted of age, sex, and *APOE* genotype, although marginally [165]. This correlation was much stronger in a study of cognitively normal elderly subjects and MCI patients; the PGS consisted of 31 SNPs (*APOE* excluded) that were strongly associated with CSF Aβ, CSF tau, Aβ deposition load, neurofibrillary tangles, and rapid longitudinal clinical progression [166]. However, not all of the results showed a significant correlation. Darst and colleagues found that pathway-specific PGS consisting of genes involved in Aβ clearance, cholesterol metabolism, and immune response cannot provide increased predictive power for CSF Aβ, neurodegeneration, and tau pathology after excluding *APOE* [167]. The inconsistent results among different studies may be because of the different SNPs used in each study and the different sample characteristics, which should be addressed in further studies.

Structural MRI and functional MRI research provided more evidence about the influence of polygenic risk on the brain. Sabuncu and colleagues used PGS consisting of 31 SNPs (including *APOE*) to investigate the relationship of polygenic risk with cortical thickness in seven AD-specific regions. They found that PGS was significantly correlated with the thickness of these regions, including the entorhinal area and part of the temporoparietal area [168]. Additionally, PGSs consisting of nine SNPs related to inflammation or immunity were reported to be significantly correlated with the thickness of the regional cortex in a group of healthy older people [169]. In some specific regions, an association between PGS and precuneus volume was found in a recent study [170]; however, the most frequently reported structural regions that were affected by PGS were the entorhinal cortex and hippocampus. Harrison and colleagues calculated PGS that represented *APOE*, *CLU*, *PICALM*, and family history, and found that both weighted and unweighted PGSs were strongly related to the change in the thickness of the hippocampus and entorhinal cortex [171]. The volume loss in these two regions was also reported in an ADNI study, and the PGS consisted of 31 SNPs in this study [172]. Lupton and colleagues once again highlighted the sensitivity of the hippocampus. They examined the association of the hippocampus and amygdaloid volumes, and the PGS consisted of 19 SNPs that did not include *APOE*. They found a significant correlation between hippocampus volume and PGS in healthy old people and in MCI patients, but not in healthy young people [173]. However, the effect of PGS on the hippocampal function of healthy young people was found in an fMRI study [174]. The influence of PGS is also reflected with white-matter disruption. Foley and colleagues not only reported lower hippocampal volume with higher PGS, even when excluding the effect of *APOE*, but also reported that the FA of the right cingulum was inversely correlated with PGS [175]. As of now, the number of neuroimaging studies on PGS risk is still insufficient, especially studies examining white matter and brain functional activity. We expect these future studies to provide a deeper understanding of the pathology of PGS through neuroimaging.

## 4. Discussion

In this review, we examined the effects of genetic factors on AD risk, cognition, and the brain. The ε4 allele of *APOE* not only increases the risk of AD, but also reduces the age of onset of AD. Behavioral studies showed that the ε4 allele is significantly associated with decreased cognitive performance (especially memory) and with cognitive decline. Imaging studies also showed that ε4 is associated with changes in the brain internal environment (Aβ deposition, CSF biomarkers, and glucose metabolism), gray-matter atrophy, white-matter damage, brain activation, and brain connectivity. The detrimental effect of the *APOE* ε4 allele in the aged was confirmed. In addition to the ε4 allele, polymorphisms within the *APOE* promoter region are also associated with the risk of AD, cognition, and brain changes. However, there are few related studies, and the promoter region also has a complex effect on the *APOE* genotype; therefore, further research is needed. In addition, some other genetic factors also interact with the *APOE* gene and produce a complex gene–gene effect. Both AD and individual cognitive function are affected by multiple genes. Considering the complex effects of different genes, PGS seems to be an appropriate method to comprehensively investigate genetic factors. This method was also applied to cognitive and brain research by increasing numbers of researchers. Next, we conduct a simple discussion based on past research and make recommendations for future research.

### 4.1. Study Sample

#### 4.1.1. Age

Age is one of the most important factors that can significantly influence the effect of the expression of *APOE* and other risk genes. The negative influence of *APOE* ε4 on cognition increases with age [176]. There is evidence demonstrating that *APOE* has no association with cognition or AD risk in the young [177] or the oldest old people [178,179]. The effects of genetics on neuroimaging are more likely to be observed at specific ages [124]. This may be because the effect of *APOE* ε4 may not be detectable when the subjects are young, and, in old age, the impairment caused by age may mask the effect of *APOE* ε4. However, there were also studies reporting a protective effect of *APOE* ε4 on cognition in children and young adults, suggesting that *APOE* ε4 carriers may have superior cognitive performance than non-carriers [180]. An “antagonistic pleiotropy” theory was used to explain this phenomenon, which represents a positive effect of a gene in early life but a negative effect of the gene later in life [181]. Superior cognitive performance in the oldest old were also reported, which makes the *APOE* ε4 allele seem protective [182]. However, researchers believe that this is due to “selective survivors”, and survivors may have a more positive effect in the absence of other AD risk factors or the existence of other protective factors [183]. In addition, the cognitive domain that was significantly affected by *APOE* ε4 differed in age. Kim and colleagues found that language, visuospatial, and frontal function were affected by APOE4 in subjects younger than 75 years, but memory was the most affected function in people older than 75. Additionally, a genotype × age interaction was also found in the same study [41]. After controlling for the status of the *APOE* genotype, the *APOE* promoter caused a decline in global cognition, memory, processing speed, and executive function, which were effects that were also age-related [66]. The gene dose effect of *APOE* improved with increasing age as well [120]. Regarding the causes of this series of phenomena, we believe aging is one of the unavoidable interfering factors. Since aging causes similar cognition and brain impairments, it is important for researchers to distinguish the effects of aging and AD genetic risk factors. Only in this way can the effect of *APOE* and other risk genes on the pathology of AD be accurately described.

#### 4.1.2. Family History

In the process of summarizing these studies, we found that the differences in family history may also influence the role of the *APOE* gene in the pathological development of AD. Bloss and colleagues found that, in children, carrying *APOE* ε4 had no effect on cognition; however, those children with both *APOE* ε4 and a family history had significantly poorer performance on cognitive tests [184]. Increased activation in the hippocampus, posterior cingulate, and temporoparietal regions was also found in older people with both *APOE* ε4 and family history [185]. However, the effect was sometimes reversed. In an episodic memory task, *APOE* ε4 carriers without a family history of AD showed increased MTL activation, but those with a family history of AD showed the least activation in the same region [186]. A family history is always strongly linked with genes. The interaction between the *APOE* gene and family history may actually be an interaction between genes. Due to the development of polygenic testing, we observed an increasing number of genes that interacted with the *APOE* gene, such as *BDNF* [71], *COMT* [70], and the *APOE* promoter [68]. However, improved models are still needed to shed more light on the relationship between family history and these risk factors.

#### 4.1.3. Other Diseases

The *APOE* gene is not the only genetic risk factor for AD; also, it is not the only gene associated with AD. Many other neurological disorders were confirmed to be affected by *APOE*. Compared to non-carriers, *APOE* ε4 carriers with traumatic brain injury had significantly increased Aβ deposition [187], and they may also suffer from poor neurological outcomes [188]. People with both *APOE* ε4 and type 2 diabetes had a significantly higher risk of AD than non-carriers [189]. However, at the same time, diabetes was associated with the non-*APOE* genotype in an AD patient group [190]. The coexistence of *APOE* ε4 and hypertension was associated with worse cognitive function compared to those with neither or either alone [191]. As exhibited by stroke, vascular dementia, multiple sclerosis, Parkinson’s disease, dementia with Lewy bodies, and so on, there are many other neurological disorders that are affected by the *APOE* gene or that can interact with the *APOE* gene [138]. It is more complicated with polygenes, and these genes may all be closely related to different diseases. Those risk genes involved in cholesterol metabolism, such as *CLU*, ATP-binding cassette subfamily A member 7 (*ABCA7*), and *SORL1*, may also be associated with hyperlipidemia; those risk genes involved in immune response, such as *CR1*, *CD33*, *MS4A*, *TREM2*, and *CLU*, may also be associated with diseases related to the immune system [192]. Therefore, the approach to identifying pathways related to the pathogenesis of AD from complex gene effects is particularly important in AD research. However, the pathological theory of AD is still controversial, and it is difficult to completely separate some diseases from AD.

### 4.2. Methodological Issue

#### 4.2.1. Uniformity

The very large discrepancy between study sample characteristics makes it particularly difficult to compare the results of different studies. The inclusion and exclusion criteria of participants, the ethnic composition of the research sample, the family history of AD of each subject, and even the sex ratio can make a difference in the results. The neuropsychological tests used in studies were sometimes different; for example, different memory tests could focus on different types of memory or different components of memory, and the reliability and validity may also differ between these tests. These factors make comparisons between studies inconclusive, although they all measure memory function. Another large difference between studies is examining polygenic risk. As we can see from Table 2, the number of SNPs used to estimate the PGSs is quite different; the number could be as low as three in one study [94] to as high as 31 in another study [172]. The criteria for obtaining SNPs are also diverse from each other; they could be defined as specific SNPs [166] or they could be determined by different thresholds [169]. Some genes such as *APOE, BIN1, CLU, ABCA7, CR1, PICALM, MS4A, CD33, TMM40*, and *CD2AP* were included in the PGSs frequently, but the pathways via which they affect AD deserve further investigation. There was also a difference in whether the *APOE* gene was included, which could make a large difference in the results. It is difficult to resolve all the differences among studies, but we at least need to be aware of these differences when we attempt to compare the results from different studies.

#### 4.2.2. Study Design

Longitudinal studies are needed. The effect of *APOE* ε4 is different depending on disease stage of AD [111], and longitudinal tracking of the same sample can more accurately describe the changes in the influence of the *APOE* gene on various biomarkers, establishing a better pathological change model. Functional MRI studies with improved designs are needed and would increase the interpretability of risk genes on specific cognitive abilities. However, there are currently few of these research designs, especially in the study of the *APOE* promoter, gene interaction, and polygenic risk factors. Some new techniques may also lead to interesting discoveries in this field, such as machine learning, virtual reality, and multimodal synchronous imaging. At the same time, researchers should be thinking about exploring the effect of non-risk alleles on risk genes, including the protective effect of *APOE* ε2 and its mechanism [195]. Some researchers found that drug interventions had better effects in people with *APOE* ε4 [196]. The mechanism of this effect and whether it also happens with other risk genes should be addressed by further intervention studies.

## 5. Conclusions

Although the precise biological changes that cause AD are still not fully revealed, genetics remains a non-negligible factor in pathogenesis. Many genes, including the *APOE* gene, are related to AD risk. Thus, integrating different genetic loci and investigating polygenic risk is reasonable. Although some researchers considered the association between polygenic risk, cognition, and the brain, the studies are limited. The influence and mechanism of polygenic risk on cognition and the brain are still undefined. The interaction between polygenic risk and other AD risk factors (e.g., age, cardiovascular disease risk factors, education, and social and cognitive engagement) also warrants further study. Continued investigations integrating polygenic risk, the brain, and cognition will move the field closer to revealing the mechanism of AD pathogenesis.

## Figures and Tables

**Table 1:** Studies of genetic association with the apolipoprotein E (*APOE*) gene.

Study	Participants	Genes	Interaction Impact on Disease	Possible Mechanisms Described by the Authors
Martinez et al., 2009 [70]	223 MCI patients, 345 AD and 253 HC	*COMT*	*COMT* (Val158 Met) polymorphism is not an independent risk factor for AD or MCI, but shows a synergistic effect with *APOE* ε4 allele that proves greater in women with AD.	Lowering the estrogen levels of brain.
Wang et al., 2005 [75]	66 AD and 86 HC	*COMT*	The *COMT* high-activity genotypes and APOEε4 allele had a synergistic effect on the risk of AD.	A high metabolism of estrogen by *COMT* may have reduced the protective effect of estrogen in AD.
Sapkota et al., 2017 [76]	634 non-demented older adults	*COMT BDNF*	*APOE* ε4+ carriers with *BDNF* Met/Met genotype and increasing allelic risk in the *COMT* + *BDNF* risk panel had poorer executive function performance.	−
Ward et al., 2014 [71]	433 older adults (50–79 years)	*BDNF*	In *BDNF* Val homozygotes, the cognitive consequences of *APOE* polymorphisms were minimal. However, in *BDNF* Met carriers, the hypothesized beneficial/detrimental effects of *APOE* polymorphisms were found.	Firstly, there is a biological interaction related to the systems or aging-related roles of the encoded proteins. Secondly, the additive effects of the polymorphisms caused the analyses to reach statistical significance.
Gomar et al., 2016 [72]	175 healthy subjects and 222 with prodromal and established AD	*BDNF*	*BDNF* Met and *APOE* ε4 carriers had thinner posterior cingulate and precuneus cortices in healthy subjects, and longitudinal decline in entorhinal thickness in MCI and AD.	−
Persson et al., 2013 [73]	888 non-demented adults (35–85 years)	*BDNF*	A joint effect on memory decline in *BDNF* × *APOE* × age, with the subjects carrying the Met allele, as well as at least one copy of the *APOE* ε4 allele showing magnified effect sizes with increasing age on memory decline, while the homozygote Val subjects carrying the ε4 allele showed a decreased slope.	−
Yu et al., 2007 [77]	193 late-onset AD, 232 subjects with no cognitive impairment, and 125 individuals with other neurodegenerative disorders	*TOMM40*	It showed intriguing linkage disequilibrium with the ε4 allele and was strongly associated with the risk for developing late onset AD.	−
Roses et al., 2009 [78]	191 AD and 131 HC (mean age: about 75 years)	*TOMM40*	Individuals with long poly-T repeats linked to *APOE* ε3 develop late onset AD on an average of 7 years earlier than individuals with shorter poly-T repeats linked to *APOE* ε3.	It is possible that the rs10524523 polymorphism, alone or in conjunction with other single-nucleotide polymorphisms in *TOMM40*, acts at a distance to affect transcription of *APOE*.
Johnson et al., 2011 [80]	117 healthy APOE ε3 homozygous adults (mean age: about 55 years)	*TOMM40*	Those who were homozygous for very long poly-T lengths had poorer memory than those who were homozygous for short poly-T length in *APOE* ε3/3.	−
Yu et al., 2017 [81]	1151 old people (mean age: about 78.5 years)	*TOMM40*	It revealed an association of *APOE* ε3/3-*TOMM40*′523 haplotypes with cognitive decline in community-based older persons such that the S/S poly-T genotype is related to faster cognitive decline, primarily in the domains of episodic and semantic memory.	The *TOMM40* variant is implicated in affecting the level of neurofilament light proteins in cerebrospinal fluid.
Louwersheimer et al., 2017 [82]	A family with 9 AD patients spanning 4 generations, with an inheritance pattern suggestive of autosomal dominant	*SORL1*	All four affected family members carried a rare variant in the vacuolar protein sorting domain 10 domain of the *SORL1* gene, associated with Aβ protein precursor processing and AD risk.	A combination of homozygous or heterozygous *APOE*ε*4* and dysfunctional *SORL1* may lead to abnormal increases in extracellular Aβ loads.
Barral et al., 2012 [83]	1365 subjects in the National Institute on Aging Late-Onset Alzheimer’s Disease Family Study	*CR1*, *BIN1*, *CLU*, *PICALM*	Several genotype patterns influenced episodic memory performance.	−
Gharesouran et al., 2014 [84]	160 patients with late-onset AD and in 163 HC	*PICALM*, *BIN1*	The associations with *PICALM* and *BIN1* were only significant among subjects without the *APOE* ε4 allele.	−
Keenan et al., 2012 [85]	1709 subjects (697 deceased) from the Religious Orders Study and the Rush Memory and Aging Project	*CR1*	A significant interaction between our candidate functional variant rs4844609 and the presence or absence of *APOE* ε4 on episodic memory decline.	−
Liao et al., 2014 [86]	536 AD cases and 307 cognitive-intact elder controls	*ABCA7*	The influence of *ABCA7* was only evident in individuals without *APOE* ε4 alleles but absent in ε4 carriers.	−
Casati et al., 2018 [87]	57 MCI, 50 AD, and 42 non-demented healthy subjects (mean age: about 78.5 years)	*TREM2*	Higher *TREM2* levels in allele ε4 of apolipoprotein E carriers than non-carriers in MCI and particularly in MCI-AD.	The upregulation of *TREM2* could be a mechanism to counteract neuroinflammatory processes in MCI patients who progress to AD.
Espeseth et al., 2006 [157]	230 healthy middle-aged (53–64 years) and older (65–75 years) adults	*CHRNA4*	*APOE*-ε4 carriers who were also *CHRNA4* TT homozygotes showed disproportionately slowed reaction time (RT) following invalid location cues. There was also a trend for individuals with combined *APOE*-ε4/*CHRNA4* TT genotypes to show both lower white-matter volume and slower overall RT on the attention task.	It remains for further research to determine which of several underlying mechanisms—acetylcholine synthesis, cholinergic neuronal metabolism, synaptic availability of acetylcholine, the affinity of cholinergic receptors, or other factors—are responsible for the interactive effects of *APOE* and *CHRNA4* on attention.
Morgen et al., 2014 [158]	165 patients with early AD dementia	*PICALM*	There was a synergistic adverse effect of homozygosity for the *PICALM* risk allele G in rs3851179 and *APOE* ε4 on volume in prefrontal and performance on the Trail Making Test.	The *APOE* and *PICALM* risk genotypes may contribute to Aβ accumulation through different mechanisms, ultimately leading to synaptic dysfunction and loss.
Thambisetty et al., 2013 [159]	57 non-demented older individuals (mean age: about 78.5 years) and 22 cognitively normal older individuals (mean age: about 77.1 years)	*CR1*	Carrying a risk allele of the *CR1* rs3818361 results in a reduced brain amyloid burden compared to non-carriers, but only in ε4 non-carriers.	The *CR1* risk allele might modify the relationship between *APOE* genotype and brain amyloid deposition.
Liu et al., 2018 [160]	710 individuals (mean age: about 65 years)	*SPON1*	Significant *SPON1* × *APOE* genotype interactions in working memory and executive function performances. The effects of ε4 allele on activation of right inferior frontal gyrus, triangular part were modulated by rs2618516 in a working memory task.	−
Shen et al., 2017 [161]	287 healthy, young, right-handed subjects (mean age: 22.7 ± 2.4 years, ranging from 18 to 29 years)	*SORL1*	Significant *SORL1* × *APOE* non-additive interaction was found in negative resting state functional connectivity (rsFC) between the hippocampus and inferior frontal gyrus. Compared with subjects with TT genotype, *SORL1* G-allele carriers had a stronger negative rsFC in *APOE* ε4 carriers, but a weaker negative rsFC in *APOE* non-ε4 carriers.	−
Zhang et al., 2017 [162]	267 healthy young adults (mean age: about 22.8 years)	*KIBRA*	Epistatic effects showed *APOE* × *KIBRA* interaction in the functional connectivity density (FCD) of the dorsolateral prefrontal cortex (DLPFC). The FCD of the DLPFC showed *APOE* risk-allele-dependent reduction (ε2 > ε3 > ε4) in *KIBRA* TT homozygotes, but *APOE* risk-allele-dependent increase in *KIBRA* C-carriers.	One candidate explanation for the complex *APOE*–*KIBRA* interactions on brain FCD may be the differential effects of genetic variations in *APOE* and *KIBRA* on the long-term potentiation of memory-related brain regions.
Porter et al., 2018 [163]	602 CN adults	*KIBRA*	In comparison to *APOE* ε4- individuals carrying the rs17070145-T allele, significantly faster rates of cognitive decline, and hippocampal atrophy were observed in individuals who were *APOE* ε4+ and did not carry the rs17070145-T allele.	Synaptic plasticity, which is altered in AD, is modulated by dendrin, which in turn binds to the protein that *KIBRA* encodes.

HC, healthy control; CN, cognitive normal; MCI, mild cognitive impairment; AD, Alzheimer’s disease.

**Table 2:** Studies of polygenic risk on cognition and brain.

Study	Participants	Study Design	SNP	*APOE*	Conversion Risk	Cognitive Impact	Neuroimaging Impact
Sabuncu et al., 2012 [168]	104 CN (75.9 ± 5.1) and 100 AD (75.1 ± 7.8)	Cross-sectional study	26	N		The PGS was significantly associated with CDR-SB, MMSE, and AD diagnosis.	AD-specific cortical thickness was correlated with the PGS, even after controlling for *APOE* genotype and CSF levels of Aβ_42_. The association remained significant in CN subjects with levels of CSF Aβ_42_ in the normal range and in *APOE* ε3 homozygotes.
Rodriguez-Rodriguez et al., 2013 [90]	228 MCI	Longitudinal study (26.3 months)	8	N	PGS was not associated with risk of conversion from MCI to AD. MCI-converters to AD harboring six or more risk alleles progressed twofold more rapidly to AD when compared with those with less than six risk alleles.		
Verhaaren et al., 2013 [91]	Non-demented 5171 (age range 45–99)	Cross-sectional study	12	Y		PGS was primarily associated with memory.	
Marden et al., 2014 [92]	10401 (memory score sample), 7690 (AD probability scores) non-Hispanic white and black	Cross-sectional study	10	Y	Each 0.10 unit change in PGS was associated with larger relative effects on dementia among aged 65+.	Each 0.10 unit change in the PGS was associated with a −0.07 standard deviation difference in memory score among aged 50+.	
Carrasquillo et al., 2015 [99]	CN 2674	Longitudinal study	10	Y	PGS was associated with progression to MCI/LOAD.	PGS was associated with worse memory.	
Martiskainen et al., 2015 [164]	890 AD (69.8 ± 8.2) and 701 CN (69.1 ± 6.2)	Cross-sectional study	22	Y/N			PGS associated with CSF Aβ_42_ levels in the clinical cohort, and with soluble Aβ_42_ levels and γ-secretase activity in the neuropathological cohort. The γ-secretase effect was independent of *APOE*.
Xiao et al., 2015 [94]	459 AD (71.2 ± 9.6), 751 CN (72.7 ± 5.9) Chinese	Cross-sectional study	3	N	PGS significantly associated with AD risk.		
Sleegers et al., 2015 [89]	1162 AD (74.4 ± 8.9) and 1019 CN (76.2 ± 8.5)	Cross-sectional study	22	Y	Risk of AD increased with PGS; onset age decreased with increasing PGS.		CSF Aβ_42_ decreased with increasing PGS.
Andrews et al., 2016 [98]	Non-demented 1689 (62.54 ± 1.51)	Longitudinal study	12	Y		PGS was associated with worse performance on episodic memory.	
Harrison et al., 2016 [171]	66 baseline participants (63.0 ± 10.4) and 45 follow-up participants (63.2±7.8)	Longitudinal study (2 years)	21	Y			Both unweighted risk score and weighted risk score correlated strongly with the percentage change in thickness across the whole hippocampal complex, driven by a strong relationship to entorhinal cortex thinning. By contrast, at baseline, the risk scores showed no relationship to thickness in any hippocampal complex subregion.
Louwersheimer et al., 2016 [97]	1730 MCI from 4 independent datasets	Longitudinal study	18	N		PGS was modestly associated with cognitive decline over time.	PGS was modestly associated with CSF levels of tau and p-tau.
Lupton et al., 2016 [173]	1674 older (aged >53 years; 17% AD, 39% MCI) and 467 young (16–30 years) adults	Cross-sectional study	Different thresholds	N			PGS associated with reduced hippocampal volume in older CN and MCI. No associations were found in young adults.
Marden et al., 2016 [93]	8253 non-Hispanic whites and blacks	Longitudinal study	22	Y/N		PGS can predict a more rapid decline in memory in whites and blacks; PGS without *APOE* ε4 only can predict memory decline in whites.	
Darst et al., 2017 [167]	1200 at baseline (53.6 ± 6.6)	Longitudinal study	21	Y		Non-significant for associations between the PGS and cognitive outcomes.	These additional variants did not add much predictive power over *APOE* alone on biomarkers of Aβ deposition, neurodegeneration and tau pathology.
Desikan et al., 2017 [172]	More than 80,000 people from two projects	Longitudinal study	31	N	ADGC Phase 1: highest PGS quartile, lower age onset and the highest yearly AD incidence rate. *APOE* ε3/3 individuals: PGS modified expected age of AD onset by more than 10 years between the lowest and highest deciles. Independent cohorts: PGS strongly predicted empirical age of AD onset and longitudinal progression.		PGS was associated with neuropathology (Braak stage of neurofibrillary tangles and Consortium to Establish a Registry for Alzheimer’s Disease score for neurotic plaques) and in vivo markers of AD neurodegeneration (volume loss within the entorhinal cortex and hippocampus)
Foley et al., 2017 [175]	272 T1 (24.8 ± 6.9), 197 DTI (23.9 ± 5.1), 87 Hopkins Verbal Learning Task (23.9 ± 4.4)	Cross-sectional study	7 thresholds	Y/N			A significant association between PGS and left hippocampal volume; this effect remained when the *APOE* gene was excluded. The fractional anisotropy of the right cingulum was inversely correlated with PGS.
Lacour et al., 2017 [96]	4 MCI groups 853/812/1245/306	Longitudinal study	9	N	PGS predicted a small effect on the risk of MCI to AD progression in *APOE* ε4 carriers.		
Voyle et al., 2017 [165]	About 250 people with normal and abnormal CSF Aβ from ADNI	Cross-sectional study	−	N			A case/control PGS is marginally more predictive of Aβ and tau pathology than the basic models (with age, gender and *APOE* genotype).
Xiao et al., 2017 [174]	231 CN (age range 19–55)	Cross-sectional study	6 thresholds	N		Almost no significant association of PGS with cognition.	There was a significant negative relationship between PGS and hippocampal function.
Ge et al., 2018 [104]	702 participants (221 CN, 367 MCI, and 114 AD) and a subset of 669 participants	Longitudinal study	Different thresholds	N			Only weak associations between PGS and baseline Aβ were present. PGSs were associated with hippocampal atrophy in Aβ− and weakly associated with baseline hippocampal volume in Aβ+.
Kauppi et al., 2018 [193]	336 MCI (baseline age range 55–89)	Longitudinal study (3 year)	31	Y	PGS significantly predicted time to progression from MCI to AD over 120 months, and PGS was significantly more predictive than *APOE* alone.	PGS improved the prediction of change in the CDR-SB score and MMSE over 36 months in MCI at baseline, beyond both *APOE* and baseline levels of brain atrophy.	
Li et al., 2018 [170]	360 CN (19.4 ± 1.1) in discovery dataset and 323 CN (22.7 ± 2.5) in replication dataset	Cross-sectional study	−	Y/N		No correlation between PGS and any cognitive measure in either sample.	In both cohorts, an elevated PGS was associated with a smaller precuneal volume, and the effect remained after excluding the *APOE* genotype.
Lin et al., 2019 [194]	2907 stroke-free individuals (76.73 ± 5.83)	Cross-sectional study	3 thresholds	Y/N			PGSs were associated with lobar cerebral microbleeds, white-matter lesion load, and coronary artery calcification, mostly explained by single-nucleotide polymorphism in the *APOE* region. The effect of PGS on cognition was partially but significantly mediated by cerebral microbleeds, white-matter lesions, and coronary artery calcification.
Tan et al., 2018 [166]	347 CN (baseline age range 59.7–90.1), 599 MCI (baseline age range 54.4–91.4), and 485 (age at death range = 71.3–108.3) in another cohort	Longitudinal study	31	N			Even after accounting for *APOE* ε4 effects, PGS may be useful in MCI and preclinical AD therapeutic trials to enrich for biomarker-positive individuals at highest risk for short-term clinical progression.

CN, cognitive normal; MCI, mild cognitive impairment; AD, Alzheimer’s disease; PGS: polygenic risk score; Y, *APOE* included in PGS; N, *APOE* not included in PGS; Y/N, Both situations of *APOE* included and not in PGS; CDR-SB, Clinical Dementia Rating Sum of Boxes; MMSE, Mini-Mental State Examination; CSF, cerebrospinal fluid; ADGC, Alzheimer’s Disease Genetics Consortium.
